# Screening and risk factor analysis of osteoporosis in elderly patients with chronic heart failure

**DOI:** 10.1186/s40001-025-03665-2

**Published:** 2026-01-03

**Authors:** Tiantian Tang, Namin Ma, Shilin Yu, Shijie Peng, Wenyan Liu, Yahui Geng

**Affiliations:** Department of Endocrinology, Beijing Geriatric Hospital, Beijing, China

**Keywords:** Elder, Heart failure, Osteoporosis, Risk factors

## Abstract

**Objective:**

To evaluate osteoporosis in hospitalized elderly patients with chronic heart failure and analyze its risk factors.

**Methods:**

A total of 230 elderly patients with heart failure from October 2021 to October 2022 were included in this study. Bone mineral density (BMD) was measured via dual-energy X-ray absorptiometry, and the incidence of osteoporosis was analyzed. *T* tests or *Χ*^2^ tests were used to compare the influence of each factor on the detection rate, and multivariate logistic regression analysis was used to analyze the independent risk factors.

**Results:**

The prevalence of osteoporosis was 61.95% in 205 elderly patients with heart failure. The lower the BMI is, the worse the heart function, and the lower the score of daily activity, the higher the detection rate of osteoporosis in heart failure patients; the incidence of osteoporosis was greater in patients with heart failure accompanied by diabetes, cognitive impairment, chronic kidney disease, and malnutrition (*P* < 0.05). Multiple logistic regression analysis revealed that BMI, activities of daily living, diabetes mellitus, and malnutrition were independent risk factors (*P* < 0.05).

**Conclusion:**

Osteoporosis is common in hospitalized elderly patients with heart failure. Screening for osteoporosis should be carried out as early as possible to intervene in risk factors in time. Poor nutritional status, low body weight, decreased living ability, and diabetes mellitus were the independent risk factors for osteoporosis (*P* < 0.05).

## Background

Heart failure is the final stage of various heart diseases. With the increasing aging of society, chronic heart failure has become a major public health problem. Heart failure is a chronic multisystem disease closely related to different metabolic disorders, which affect the skeletal muscle system and lipid metabolism; leads to weight loss; the loss of bone, muscle, adipose tissue, and other body components; and eventually leads to cachexia [1–3]. The most prominent bone metabolism disorder is osteoporosis, which is characterized mainly by a reduction in bone mass and degeneration of the bone microstructure, increasing the fragility of the bone and increasing its susceptibility to systemic bone disease. The incidence rates of osteoporosis and heart failure are highly correlated with age [4]. Moreover, osteoporosis is considered an independent risk factor for cardiovascular disease [5]. In our clinical practice, we have found that patients with chronic heart failure are more prone to osteoporosis. Therefore, we designed this study to investigate the detection rate of osteoporosis in hospitalized elderly heart failure patients and the potential risk factors that may contribute to osteoporosis in these patients.

In recent years, chronic diseases combined with osteoporosis have attracted increasing interest from researchers; existing studies mostly focus on research about diabetes and osteoporosis [6], or atrial fibrillation and osteoporosis or hip fractures [7], but few studies have investigated bone metabolism in elderly patients with heart failure. In this study, dual-energy X-ray was used to screen for osteoporosis in elderly patients with heart failure, and the detection rate and risk factors for osteoporosis were analyzed. This study aims to remind clinicians to screen for osteoporosis earlier, then they can initiate relevant treatments promptly, reduce the risk of fractures, and improve the prognosis of heart failure.

## Object and method

### Object

A total of 230 patients aged ≥ 60 years with chronic heart failure admitted to our hospital from October 2021 to October 2022 were selected. The inclusion criteria were as follows: (1) aged ≥ 60 years; (2) met the diagnostic criteria for chronic heart failure. The exclusion criteria were as follows: (1) patients with unstable, critical, or terminal disease; (2) myocardial infarction occurring within the last 3 months; (3) acute left heart failure caused by any cause; (4) recently used steroid hormones, vitamin D, calcium preparations, and other drugs that interfere with bone metabolism; (5) previous thyroid or parathyroid disease; (6) incomplete clinical data; (7) patients or family members who refused to participate in the study.

According to bone mineral density (BMD) levels, elderly chronic heart failure patients were divided into an osteoporosis group (test) and a nonosteoporosis group (control).

This study was approved by the hospital Medical Science Ethics Committee.

### Method

#### Data collection

Demographic and disease-related data were collected from hospitalized patients. The indicators included age, sex, smoking and drinking history, course of disease, body mass index (BMI), other chronic diseases, cardiac function grade, heart color ultrasound ejection fraction, etc. The patients were evaluated for cognitive impairment through the Mini-Mental State Examination (MMSE) with a total score of 30, and a score < 27 was classified as cognitive dysfunction. Daily activity ability was scored by the Barthel index scale (100 points for self-care, 61–99 points for mild impairment, able to complete part of daily activities independently but with certain help; moderate disability 41–60, requiring considerable assistance to complete activities of daily living). Severe dysfunction, less than or equal to 40, unable to perform most daily activities or requiring complete care.

Nutritional status was assessed via the NRS-2002 screening system, with a score of ≥ 3 indicating that the patient was at risk of malnutrition and then professional doctors from the nutrition department analyzed the patients’ comprehensive conditions to determine whether they have malnutrition. Parathyroid hormone (PTH), serum calcium, and serum 25(OH) D levels were also measured by our hospital’s laboratory.

#### Diagnosis of chronic heart failure

According to the recommended criteria of the European Society of Cardiology Heart Failure Guidelines 2021, heart function was classified into grades I to IV via the New York College of Cardiology (NYHA) grading method.

#### Screening methods for osteoporosis Bone mineral density measured by dual-energy X-ray absorptiometry (DXA)

Bone mineral density is usually expressed as a T-score, which = (measured value—peak bone mineral density of healthy young people of the same race and sex)/standard deviation of peak bone mineral density of healthy young people of the same race and sex. According to the diagnostic criteria recommended by the WHO, BMD values > 2.5 SD below the mean peak BMD (T-score ≤ − 2.5) are confirmed as osteoporosis. A reduction in bone mineral density meets the diagnostic criteria for osteoporosis, and the combination of one or more fragility fractures is considered severe osteoporosis [8].

### Statistical processing

SPSS 22.0 was used for statistical analysis. The measurement data are expressed as the means ± standard deviations (x quasi difference), and a *t* test was used for intergroup comparisons. The statistical data are expressed as percentages, and the *χ*^2^ test was used for comparisons between groups. Multivariate logistic regression analysis was used to determine the independent risk factors for osteoporosis in elderly patients with chronic heart failure, and the odds ratio (OR) and 95% confidence interval (CI) were calculated. All tests were two-sided, and *P* < 0.05 was considered statistically significant.

## Results

### Case collection

A total of 230 elderly hospitalized patients with heart failure were included. After the exclusion of 10 patients with acute heart failure, 5 patients who had recently taken drugs that could affect bone metabolism, 2 patients in critical condition, 3 patients with previous thyroid disease, and 5 patients with incomplete clinical data, 205 patients were finally included, including 91 males with an average age of 71.87 years and 114 females with an age of 78.54 years (*P* < 0.001). Among these patients, 127 had osteoporosis, and the detection rate of osteoporosis was 61.95% (127/205).

### Comparison of basic clinical data between the osteoporosis group and the nonosteoporosis group of hospitalized elderly patients with heart failure

Patients were divided into an osteoporosis group and a nonosteoporosis group according to their bone metabolic status. The basic clinical data (age, disease course, sex, smoking history, drinking history, BMI, daily activity score, functional class of NYHA) of the two groups were compared. There were significant differences in the sex ratio and smoking, drinking, BMI, daily activity ability, and cardiac function grades between the two groups (*P* < 0.05); there were no significant differences in the age and course (*P* > 0.05), as shown in Table 1.

**Table 1:** Comparison of general data and disease spectra between the two groups

Characteristics	Control	Test	*t*/*χ*2	*P* value
Age, y [(x ± s)]	74.2 ± 9.4	78.9 ± 7.7	− 3.871	0.056
Female [N (%)]	52 (65.8)	108 (85.7)	11.214	0.001
Course, y[(x ± s)]	4.36 ± 4.14	4.51 ± 4.60	− 0.282	0.779
Smoking, [N (%)]	45 (57.0)	50 (39.7)	5.83	0.016
Drinking, [N (%)]	27 (34.2)	18 (14.3)	11.214	0.001
Body mass index, kg/m^2^	21.94 ± 2.86	20.60 ± 2.44	3.583	< 0.001
Daily activity score	80.41 ± 23.07	41.40 ± 32.30	9.339	< 0.001
Functional class of NYHA	2.33 ± 0.957	2.93 ± 0.812	4.799	< 0.001

### Comparison of biochemical indices of bone metabolism between the osteoporosis group and nonosteoporosis group in elderly hospitalized patients with heart failure

The biochemical indices of bone metabolism (blood calcium, parathyroid hormone, the creatinine level, osteocalcin, and 25(OH) vitamin D) in the osteoporosis group and the nonosteoporosis group were compared, and the creatinine level in the osteoporosis group was significantly greater than that in the nonosteoporosis group (*P* < 0.05), as shown in Table 2. However, no clinically significant differences were found in other indicators between the two groups.

**Table 2:** Comparison of bone metabolism and biochemical indices between the two groups

Characteristics	Control	Test	*t*/*χ*2	*P* value
Ca, mmol/l	2.21 ± 0.15	2.18 ± 0.15	1.512	0.373
PTH, pg/ml	85.45 ± 115.22	80.77 ± 68.31	0.331	0.151
Cr, umol/l	113.31 ± 87.51	134.06 ± 105.32	− 1.414	0.034
Urea, mmol/l	9.19 ± 11.71	12.77 ± 13.36	− 1.891	0.065
Alb, g/L	38.84 ± 4.38	36.96 ± 4.23	3.050	0.910
ALP, U/L	74.34 ± 29.13	90.78 ± 58.83	− 2.310	0.181
BGP, ng/ml	22.80 ± 36.01	22.25 ± 17.81	0.130	0.133
25(OH) D, ng/ml	14.60 ± 7.69	11.94 ± 9.48	1.848	0.844

*Ca* blood calcium, *PTH* parathyroid hormone, *Cr* creatinine, *Urea* urea nitrogen, *BGP* osteocalcin, *Alb* serum albumin, *25(OH)D* 25(OH) vitamin D

### Comparison of comorbidities between the osteoporosis group and the nonosteoporosis group of elderly inpatients with heart failure

The detection rate of osteoporosis in elderly patients with heart failure combined with diabetes, CKD, malnutrition, and cognitive impairment increased, and the difference was statistically significant (*P* < 0.05), as shown in Table 3.The presence of atrial fibrillation did not affect the detection rate of osteoporosis in heart failure patients.

**Table 3:** Comparison of comorbidities between the two groups

Characteristics	Control	Test	*t*/*χ*2	*P* value
Diabetes, *N*, (%)	36 (45.6)	82 (65.1)	7.566	0.006
CKD, *N*, (%)	26 (32.9)	60 (47.6)	4.313	0.038
Malnutrition, *N*, (%)	11 (13.9)	58 (46.0)	21.943	< 0.001
AF, *N*, (%)	22 (27.8)	37 (29.4)	0.055	0.815
Cognitive disorder, *N*, (%)	5 (6.3)	46 (36.5)	23.663	< 0.001

*CKD* “Chronic Kidney Disease”, *AF* atrial fibrillation

### Multivariate logistic regression analysis of independent risk factors for osteoporosis in elderly hospitalized patients with heart failure

Multivariate logistic regression analysis revealed that decreased BMI, decreased daily activity score (D-a-s), diabetes or malnutrition were independent risk factors for osteoporosis in heart failure patients (Si < 0.05) (Table 4).

**Table 4:** Multivariate logistic regression analysis

Characteristics	B	Standard error	Wald	Si	Exp (B)	95% CI	
						Lower limit	Upper limit
Sex (M)	− 0.970	0.512	3.586	0.058	0.379	0.139	1.035
Smoking	− 0.086	0.417	0.042	0.837	0.918	0.406	2.077
BMI	0.076	0.026	8.297	0.004	1.079	1.025	1.137
Diabetes	0.776	0.375	4.287	0.038	2.172	1.042	4.528
CKD	0.191	0.461	0.172	0.679	1.210	0.491	2.986
D- a- s	− 0.034	0.007	23.402	0.000	0.966	0.953	0.980
Cr	0.002	0.002	0.953	0.329	1.002	0.998	1.007
Malnutrition	1.532	0.469	10.664	0.001	4.629	1.845	11.612

*D- a- s* daily activity score

## Discussion

Osteoporosis is an aging-related bone disease. According to the results of the first epidemiological survey on osteoporosis in China conducted by the National Health Commission in October 2018, the prevalence rate of osteoporosis in people over 65 years of age reached 32%. Heart failure is also an aging-related disease and a chronic multisystem disease. Studies have confirmed that heart failure and osteoporosis interact with each other and even share common risk factors [9]. Studies have shown that osteoporosis is a new predictor of poor prognosis in patients with heart failure [10], and heart failure also has adverse effects on osteoporosis. Patients with heart failure have poor quality of life, such as shortness of breath/sleep disturbance/weakness, which adversely affects bone metabolism, induces severe bone loss, and increases the risk of osteoporosis and fracture [11]. This study also confirmed that the prevalence of osteoporosis in the heart failure population was significantly greater than that in the general population.

The pathophysiology of bone loss in patients with heart failure has not been fully defined. People with dementia often have less activity and a poorer ability to protect themselves, leading to a loss of bone mass. Therefore, the occurrence of dementia in elderly patients with heart failure is considered a risk factor for bone loss, falls, and hip fractures [12]. This study revealed that the detection rate of osteoporosis in patients with heart failure combined with cognitive impairment significantly increased, but it failed to be concluded that cognitive impairment was an independent risk factor, which may be related to the overall age of the enrolled patients, and another possible reason is that it has a confounding effect with other risk factors mentioned earlier. In addition, the assessment of cognitive impairment is indeed affected by some subjective factors, which is also a limitation of our study.

Patients with advanced heart failure have reduced bone metabolism [13]. Our study also confirmed that decreased ability to live (decreased daily activity score), reduced mobility, and reduced outdoor exercise, which may affect vitamin D levels as a risk factor for osteoporosis in patients with heart failure. Another possible mechanism is insufficient mechanical stress stimulation reducing osteoblast activity and enhancing osteoclast activity, leading to bone loss.

Many studies have shown that the lower the body weight is, the greater the risk of osteoporosis, and a weight increase, especially the lean mass, can promote the growth of bone mass [14]. The mechanism is considered to be that weight (especially in the load-bearing part) promotes the growth of bone mass by generating load-bearing external forces on bones. Heart failure in patients due to the use of diuretic drugs or even SGLT2i drugs leads to weight loss, and in some people, long-term hypoxia and gastrointestinal congestion lead to poor appetite and weight loss, increasing the risk of osteoporosis. Our study confirms that a decrease in BMI is an independent risk factor for osteoporosis in elderly patients with heart failure.

Vitamin D deficiency is an important cause of bone mineral density loss. Vitamin D deficiency decreases calcium absorption and stimulates PTH secretion, causing bone loss. In the course of heart failure, vitamin D deficiency is more significant in people with heart failure than in healthy people because of the loss of urinary calcium due to long-term use of diuretics, reduced absorption of minerals and vitamin D due to gastrointestinal congestion, and reduced synthesis of 1–25 dihydroxy vitamin D due to liver congestion. Vitamin D deficiency is also associated with 90% of heart failure cases [15]. Our study revealed that in elderly patients with heart failure, vitamin D3 was lower in the osteoporosis group than in the nonosteoporosis group. Unfortunately, no significant difference was found, consistent with the conclusions of some previous studies [16]. However, our study confirms that malnutrition is an independent risk factor for osteoporosis in elderly patients with heart failure. Previous studies have confirmed that the incidence of malnutrition in patients with heart failure is very high [17], and malnutrition is considered a major risk factor for osteoporosis [18]. Our research results are basically consistent with previous conclusions.

The risk of osteoporosis and fracture in diabetic patients is significantly greater than that in nondiabetic patients [19]. The correlation between type 2 diabetes and BMD is affected by multiple secondary factors, and no uniform conclusion has been reached. What we already know is the use of certain antidiabetic drugs like thiazolidinediones (TZDs) can exacerbate osteoporosis. Type 2 diabetes is a risk factor for fractures associated with decreased bone mineral density, and the risk of hip fracture is 1.7 times greater than that of nondiabetic patients [20]. The most common type of diabetic macrovascular disease is cardiovascular disease; heart failure caused by cardiovascular disease is the main cause of chronic heart failure. Owing to decreased activity, the use of lipid-lowering drugs, diuretic drugs, etc., in diabetes patients with heart failure aggravates the influence of glucose metabolism disorders on bone mineral density [21], which increases the risk of osteoporosis.

## Conclusion

In summary, the incidence of osteoporosis in elderly patients with heart failure is significantly greater than that in normal elderly people. The detection rate of osteoporosis in elderly patients with heart failure combined with diabetes, CKD, malnutrition, and cognitive impairment further increases, whereas BMI decreases, life ability decreases (daily activity score decreases), and the incidence of diabetes mellitus and osteoporosis increases. Malnutrition is also an independent risk factor for osteoporosis in elderly patients with heart failure.

At present, there is no effective clinical treatment to restore normal bone mass in patients with severe bone loss, so prevention is crucial. Screening should be carried out as early as possible. This can guide clinicians to conduct a comprehensive assessment of the patients’ conditions, more rationally design their nutrition and rehabilitation plans, provide calcium supplementation for patients sooner, and avoid using medications that exacerbate bone loss. Clinicians can also timely intervene against risk factors, strictly control blood glucose, pay attention to nutritional issues, avoid excessive weight loss, encourage appropriate activities, and so on.

There are a number of limitations to this study. Owing to the small sample size and the older average age of the enrolled population, the results of this study may be biased. The research subjects of this study are indeed hospitalized elderly heart failure patients and cannot represent all elderly heart failure patients. There may be issues with sample independence between some influencing factors, such as BMI and malnutrition. However, since both are commonly used evaluation tools in clinical practice, we did not exclude them. In addition, since a rating scale was used for the assessment of cognitive impairment, there may be some subjective factors, which could affect the results. In the future, we will expand the sample size and conduct further studies on the specific mechanism.

## Data Availability

No datasets were generated or analysed during the current study.
